# Fluorescence of D-Glucose-Derived Carbon Dots: Effect of Process Parameters

**DOI:** 10.1007/s10895-023-03392-z

**Published:** 2023-08-18

**Authors:** Anna Piasek, Jolanta Pulit-Prociak, Michał Zielina, Marcin Banach

**Affiliations:** 1 Faculty of Chemical Engineering and Technology, Department of Chemical Technology and Environmental Analytics, Cracow University of Technology, Warszawska 24, 31-155 Cracow, Poland; 2 Faculty of Environmental Engineering and Energy, Department of Water Supply, Sewerage and Environmental Monitoring, Cracow University of Technology, Warszawska 24, 31-155 Cracow, Poland

**Keywords:** Carbon dots, Glucose, Fluorescence, Microwave hydrothermal synthesis, Monopotassium phosphate, Statistical analysis

## Abstract

The aim of this study was to synthesize highly fluorescent carbon dots (CDs) from glucose using a microwave hydrothermal method. It explored the impact of glucose concentration, process time, molar ratio of KH_2_PO_4_ to glucose, and homogenization time on the resulting CDs, employing a fractional plan 3^(k-1)^ with four independent parameters for twenty-seven synthesis. Results showed that longer process times at 200°C increased the fluorescence intensity of the CDs. The molar ratio of KH_2_PO_4_ to glucose, glucose concentration, and process time significantly influenced fluorescence. Homogenization was crucial for obtaining small particles, though an anti-aggregation agent might still be needed. UV-vis spectroscopy, spectrofluorimetry, and DLS were used to analyze the synthesized CDs. The UV-vis absorption maxima were observed around 230 nm and 282 nm, with peak shifts at different excitation wavelengths. Out of the twenty-seven samples, six CDs samples were identified to be below 10 nm and a total of twelve below 50 nm. Analyzing the results, the study concluded that the CDs possess strong fluorescence and are suitable for diverse applications. For enhanced fluorescence, longer process times at 200°C and the use of KH_2_PO_4_ were recommended, while shorter processes were preferred for obtaining smaller particles. Hierarchical clustering, the k-means method, Pareto charts, and profiles for predicted values and desirability were used to analyze the results. It was confirmed that higher fluorescence is favored by longer process time at 200°C and the use of KH_2_PO_4_. In order to obtain smaller particles, shorter processes should be used.

## Introduction

Carbon dots (CDs) are a new nanomaterial with many interesting properties that translate into their numerous applications. CDs are characterized mainly by high quantum efficiency, strong radiation absorption, biocompatibility, non-toxicity, stability, and ability to transfer electrons [1]. Another great advantage is that they can be obtained easily according to the principles of green chemistry. The top-down and bottom-up methods work well here. The most commonly described methods are hydrothermal, microwave, ultrasonic, solvothermal, pyrolysis, and chemical oxidation processes. CDs can be synthetic or obtained from natural raw materials or biowaste, for example, leftovers from processing fruits, vegetables, grains, eggshells, shells, and wool. Plant or animal products, such as milk, oils, honey, and juices are also used. A very good idea is to develop an effective method for obtaining CDs from used tires or waste paper [2–4]. An important feature of CDs is their easy modification, which allows the material to be customized or improved for a specific application. The use of CDs in optoelectronics, catalysis, energy storage, biomedicine, and sensors can bring many benefits [5].

Many studies devoted to CDs are based on their fluorescence (FL) [6–8]. CDs have a broad band of light excitation and a narrow band of fluorescence. They also attract attention because they emit FL of different colors depending on the position of the emission wavelength. Their FL can be controlled by adjusting the core of the structure accordingly or adding surface functional groups [9]. The main role is played by the π-electrons, as sp^2^ carbon clusters are dispersed in a carbon backbone with sp^3^ hybridization. For CDs formed by conjugating large π-domains to functional groups on the surface, the band gap formed between the domains is considered to be the source of FL. This is due to the limited size of the π-domains. The valence band is far from the conduction band. Electrons through this go to the empty state of the valence band, emitting a wave of light. Band fluorescence can be manipulated by increasing the size of the conjugated π domain, which has the effect of reducing the resulting band gap [10, 11]. There is another FL mechanism, which comes from the so-called surface defects. It refers to functional groups on the surface or sp^2^, sp^3^ hybridization of carbon. The FL emission thus induced is the result of radiation relaxation from the excited state to the ground state [2, 10].

The UV absorbance of CDs depends on their possible passivation or surface functionalization. Mostly, maximum absorption is encountered at 230-320 nm. The basic raw material for obtaining CDs is glucose, as it is a cheap reactant, is easily available, and contains a high carbon content. Furthermore, the presence of hydroxyl and carbonyl groups remaining after the decomposition of the sugar molecule deposited on the surface can increase the stability of CDs in water and induce a photoluminescence effect [4, 12]. The properties of CDs are strongly dependent on the process parameters, raw material, the pH of the reaction environment, structural modifications, or even additional compounds introduced into the reaction mixture. Such compounds are supposed to perform specific roles, such as counteracting particle aggregation. T. Yoshinaga et al. and Z. Xu et al. in their study propose the use of H_3_PO_4_ or KH_2_PO_4_ as a dehydrating agent to obtain smaller particles [12, 13].

In this work, glucose and the microwave method were used to obtain CDs. The optical properties of the particles were analyzed in detail, mainly due to their fluorescence capabilities below 300 nm of excited light. The syntheses were based on four different variable parameters, the effect of which on FL was studied. Among other things, the utility of using KH_2_PO_4_ as a dehydrating agent has been verified. The results were discussed through statistical analysis.

## Experimental

### Materials

D-(+)-Glucose (≥99.5%) from Sigma-Aldrich was used as the high-carbon raw material. In addition, KH_2_PO_4_ (≥99%) from Sigma-Aldrich was used as a dehydrating agent. Deionized water was used as the solvent in the reactions performed.

### Methods

In this experiment, the synthesis was carried out in a Magnum II microwave reactor (Ertec) at 200 °C and a constant pressure of 15 bar. This temperature was selected based on the results of preliminary experiments. Reaction mixtures with a total volume of 40 mL were placed in Teflon vessels. The experiment was planned based on the DoE (Design of Experiments) method with fractional plan 3^(k-1)^ and included 27 syntheses in deionized water with varied glucose concentration, KH_2_PO_4_:Glucose (P/G) molar ratio, process and homogenization time. The solutions were homogenized prior to synthesis for a specific time in a Hielscher UP400St homogenizer. Table 1 presents the exact parameters of each synthesis.

**Table 1:** Parameters of the variables used in the syntheses

**Sample**	**Glucose concentration (mol/L)**	**Process time (min)**	**P/G molar ratio**	**Homogenization time** **(min)**
**S1**	0.01	2	0	0
**S2**	0.01	2	5	4
**S3**	0.01	2	10	2
**S4**	0.01	20	0	4
**S5**	0.01	20	5	2
**S6**	0.01	20	10	0
**S7**	0.01	38	0	2
**S8**	0.01	38	5	0
**S9**	0.01	38	10	4
**S10**	0.05	2	0	4
**S11**	0.05	2	5	2
**S12**	0.05	2	10	0
**S13**	0.05	20	0	2
**S14**	0.05	20	5	0
**S15**	0.05	20	10	4
**S16**	0.05	38	0	0
**S17**	0.05	38	5	4
**S18**	0.05	38	10	2
**S19**	0.09	2	0	2
**S20**	0.09	2	5	0
**S21**	0.09	2	10	4
**S22**	0.09	20	0	0
**S23**	0.09	20	5	4
**S24**	0.09	20	10	2
**S25**	0.09	38	0	4
**S26**	0.09	38	5	2
**S27**	0.09	38	10	0

After synthesis, the suspension was filtered through a Carl Roth nylon syringe filter with a pore size of 0.45 μm. The effect of selected reaction variables on the fluorescence properties and parameters of the obtained products was studied. UV-vis absorption was measured using a Rayleigh UV 1800 in the 190-1100 nm range. The fluorescence of the samples was examined on a Tecan Infinite 200Pro spectrofluorometer at different wavelengths of excited light. The study was conducted using dark 96-well plates from Thermo Fisher Scientific, into which 200 μl of sample was dispensed each time. The dispensed sample plates were exposed to the selected wavelength, and the plate reader collected the data obtained from the fluorescence measurement of the samples. For better visualization of the results, images were taken when the samples were exposed to a UV lamp with a wavelength of 285 nm. The particle size was measured by DLS using a Malvern Zetasizer NanoZS apparatus using a refractive index of 2.4175 and absorption 0.001. Statistic software from StatSoft was used for statistical analysis and Origin software was used for the mathematical calculations.

## Results

### Optical Properties

**Fig. 1:**
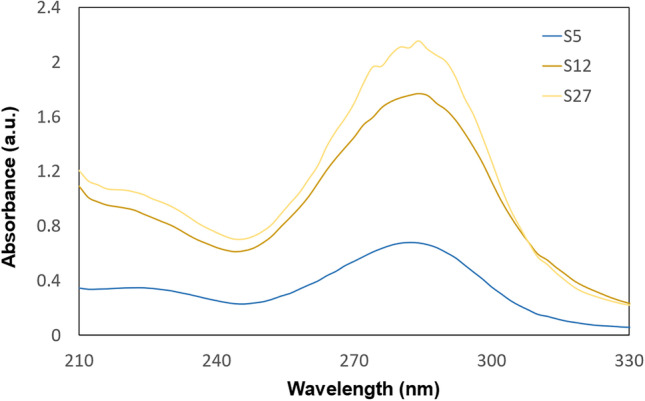
Examples of UV-vis absorption spectra of CDs with one visible peak at about 280 nm

**Fig. 2:**
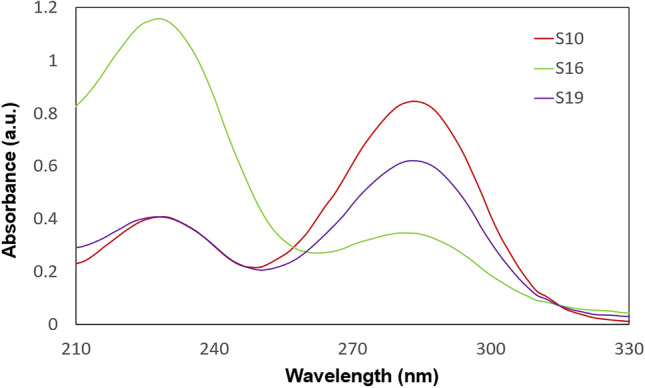
Examples of UV-vis absorption spectra of CDs with two visible peaks at about 230 nm and 280 nm

The synthesized CDs were tested for their optical properties by measuring UV-vis absorption and fluorescence intensity. CDs absorb radiation giving characteristic peaks on the spectrum at around 230 and 280 nm. The absorption at around 230 nm wavelength corresponds to the π → π* transition in the C=C bond and around 280 nm wavelength is for n → π* transition in the C=O bond [11, 14]. According to Ma et al. UV-vis absorption below 210-250 nm indicates the occurrence of which comes from the aromatic π → π* transition of C=C bonds [15]. Absorption of around 300 nm can be caused by functional groups on the CDs surface resulting from carboxyl and carboxylate functional groups [16]. Figure 1 shows the UV-vis absorption spectra for three example samples with only one peak with an absorption maximum at around 282 nm. This peak is visible on each spectrum. The peak at about 230 nm is also noticeable in some spectra, as in the examples in Fig. 2. These peaks are broad and clearly visibly on the spectrum, especially for samples obtained without KH_2_PO_4_.

**Fig. 3:**
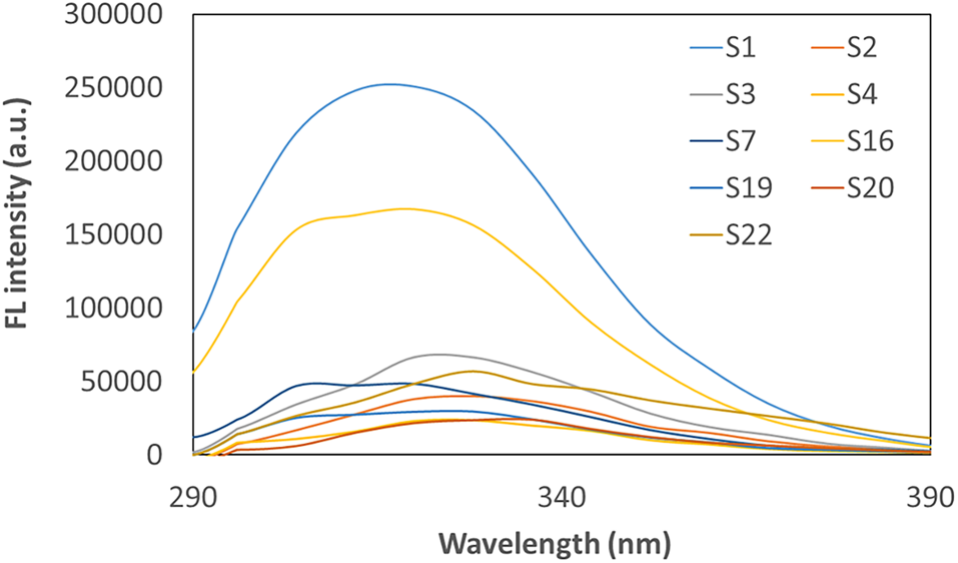
FL spectra of the obtained CDs at an excitation wavelength of 256 nm

Fluorescence analysis of the obtained CDs at 256 nm and 282 nm excitation wavelengths was carried out. Figure 3 shows the FL emission spectra at an excitation wavelength of 256 nm for the samples with the highest values. The S1 and S16 samples have the best FL properties, emitting wavelengths of light with the highest intensity in the 290-390 nm range. The maximum peak is around 320 nm. In this case, the FL spectra of individual samples differ significantly from each other, indicating that suspensions with different properties were obtained. The FL intensity for the most luminous samples (S1, S2, S3, S4, S7, S16, S19, S20, S22) is in the range of about 24,000 - 250,000 a.u. The intensity of FL is influenced, among other things, by the structure of the obtained CDs and the parameters of the synthesis carried out. The final product is a suspension of CDs, which will vary in the number of products obtained. Fluorescence increases in relation to the concentration of the substance only up to a certain value, and then rapidly decreases [17, 18]. A higher FL intensity may indicate that more CDs are obtained. The other CDs not shown on the chart had a weak FL with intensities in the range of about 3,000 - 14,000 a.u.

**Fig. 4:**
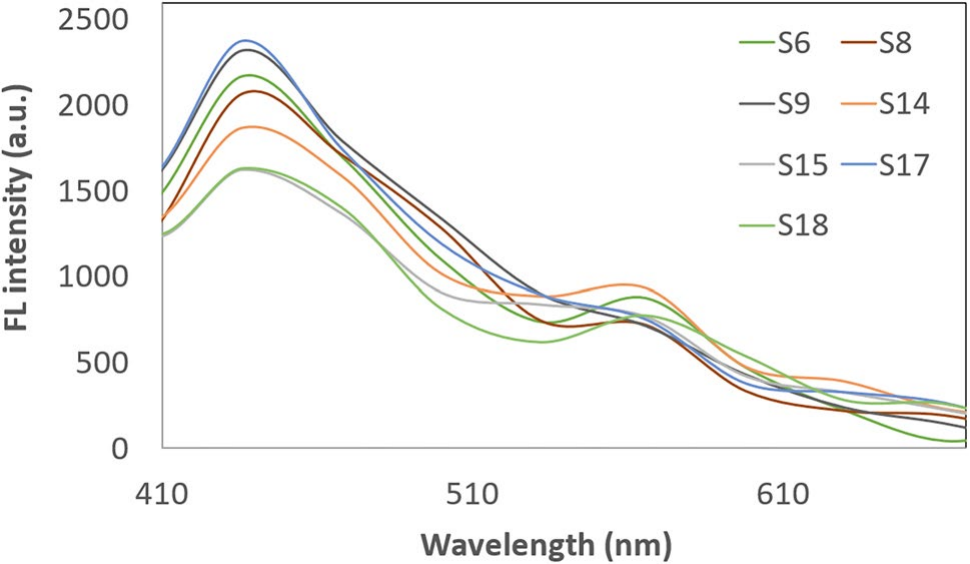
Fluorescence spectra for CDs with the highest fluorescence intensity (excitation at 282 nm)

Figure 4 shows the FL emission spectra at an excitation wavelength of 282 nm for the samples with the best fluorescence properties. The emission consists of two peaks that have maxima at about 440 and 560 nm. These samples show secondary fluorescence. Samples that emit light at an excitation wavelength of 256 nm did not show FL when excited with a wavelength of 282 nm, for example samples S1, and S16. The intensity of the emitted CDs light is much higher for the 256 nm excitation wave than for the 282 nm. Carbonaro et al. studied CDs obtained in an aqueous environment by microwaving in various combinations of modifications. They compared the properties of bare CDs with nitrogen-doped CDs. They analyzed the fluorescence properties with excitation in the 250-500 nm range. Excitation with 250 nm light gives light emissions at a maximum of about 450 nm, while excitation with 275 nm gives similar results. In an aqueous environment, there is little shift from wavelength at fluorescence peaks [19].

**Fig. 5:**
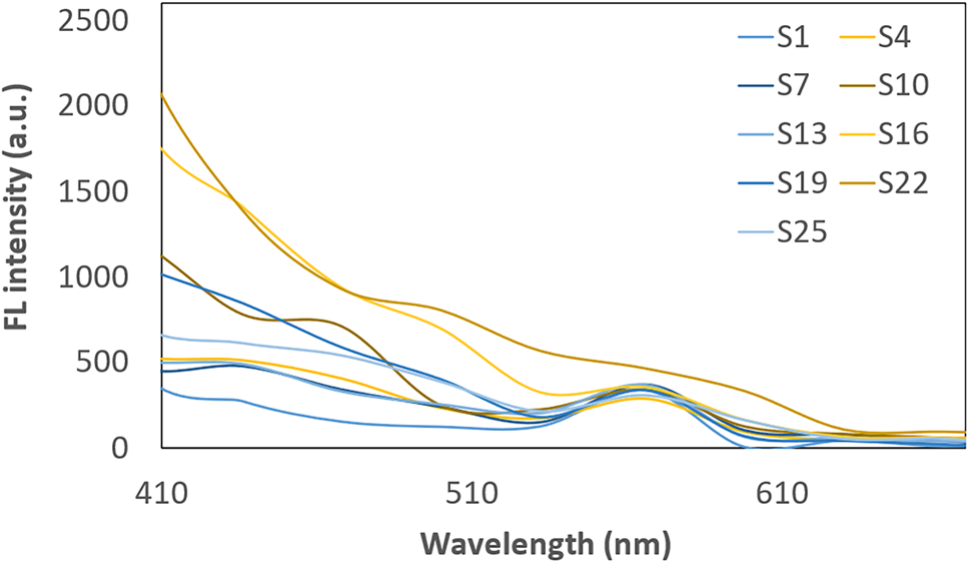
Fluorescence spectra for CDs with the lowest fluorescence intensity (excitation at 282 nm)

Samples with weak fluorescence at 282 nm illumination are highlighted in a separate graph in Fig. 5. These samples were obtained without a dehydrating agent. The FL spectra of the phosphate-containing samples do not show much difference among themselves and differ significantly from the others. In these spectra, the first peak is very indistinct and the second peak has an intensity of less than 500 a.u. The addition of KH_2_PO_4_ increases the FL intensity in the spectrum to about 2400 a.u. It can be concluded that potassium phosphate has a definite effect on the FL intensity of the obtained CDs. The use of KH_2_PO_4_ results in the embedded of a phosphate residue into the surface of CDs [20]. Such modification allows for better fluorescence properties.

**Fig. 6:**
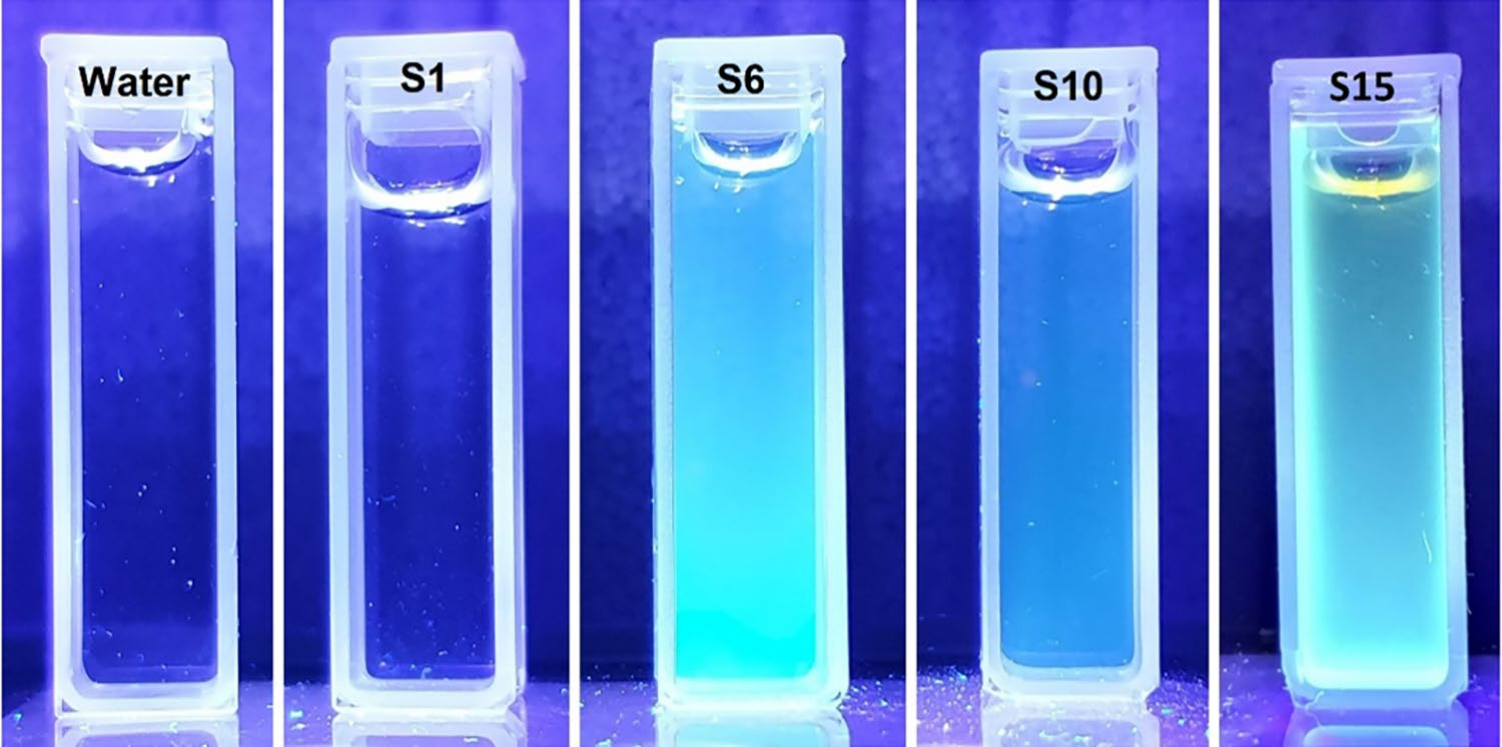
Photos of selected CDs samples exposed to a 285 nm lamp

Figure 6 shows photos of selected samples taken in the darkroom under illumination with a 285 nm UV lamp. Water is the reference sample. The fluorescence color varies from blue to blue-green. Illumination of the samples with this wavelength confirmed the results obtained in Figs. 4 and 5. S1 does not emit radiation at this excitation value, and S10 gives little fluorescence. S6 and S15 reached the high fluorescence intensity seen in Fig. 4 and also emitted strong radiation in the darkroom.

### Particle Size

**Fig. 7:**
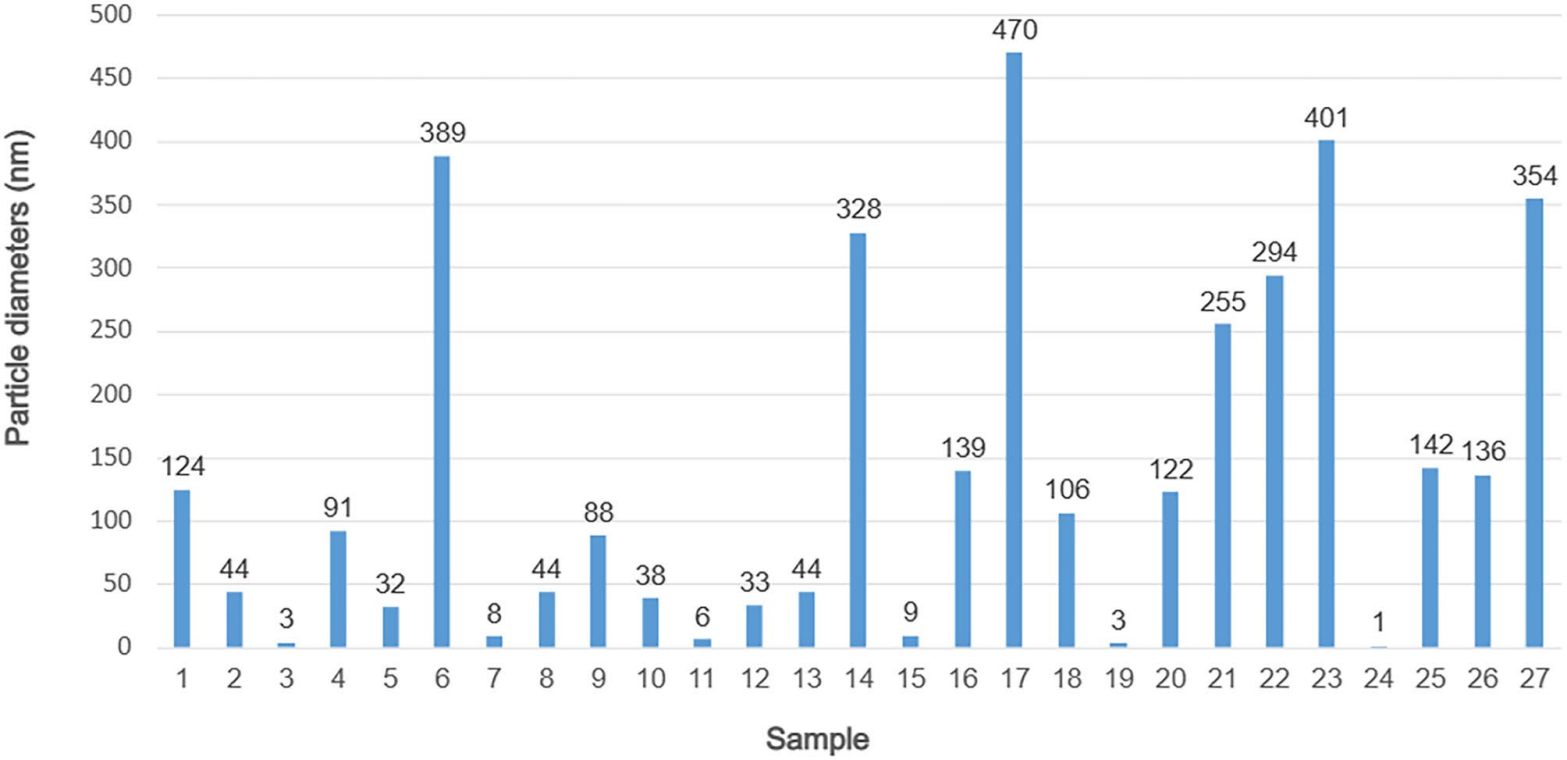
The average diameters of the particles

**Fig. 8:**
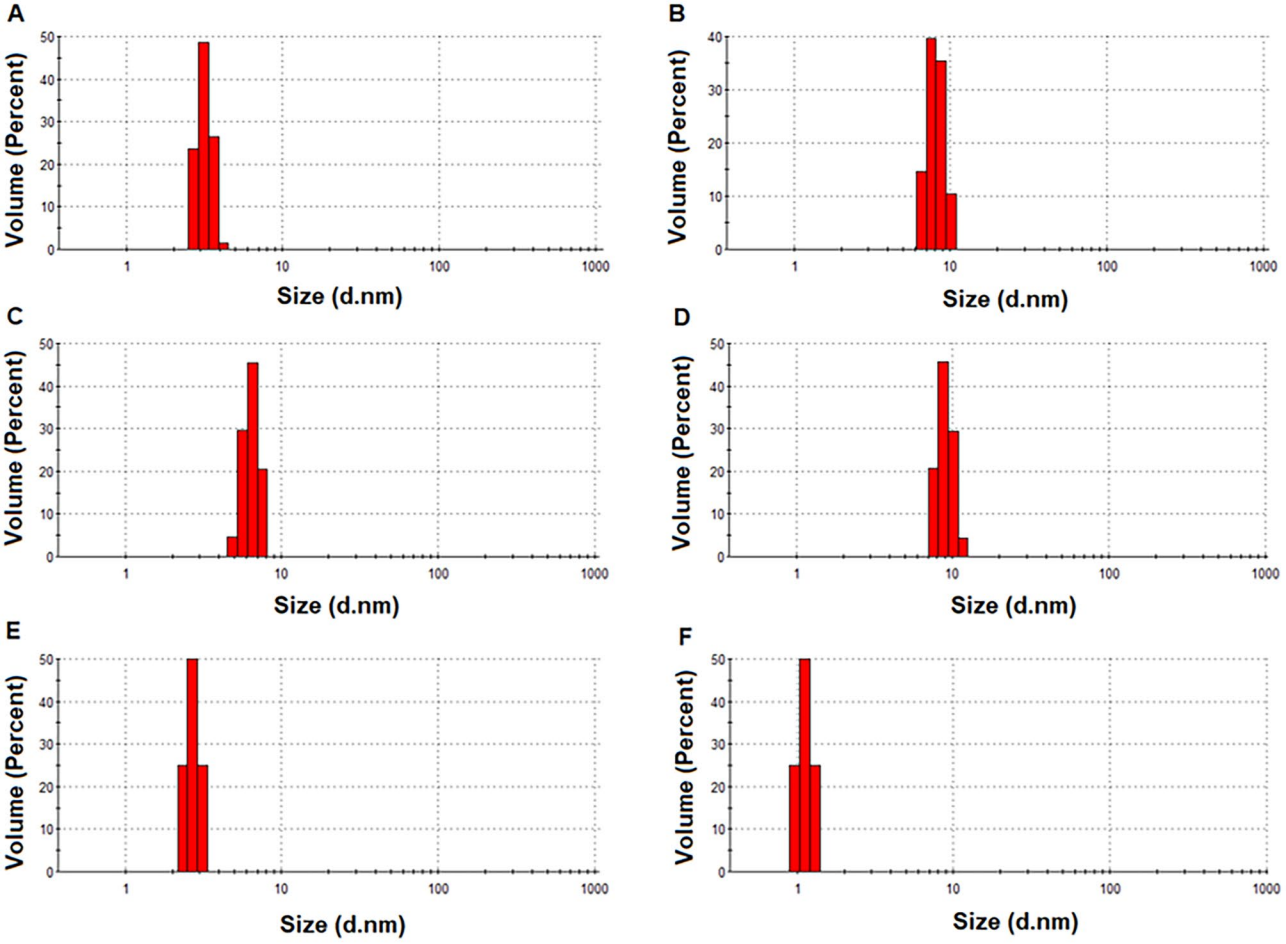
Example histograms of the particle size distribution versus volume in sample (**A**) 3, (**B**) 7, (**C**) 11, (**D**) 15, (**E**) 19, and (**F**) 24

As a result of the syntheses, particles of various sizes were obtained, which are in the range of about 1 to 500 nm (Fig. 7). Most samples of obtained CDs had a diameter of up to 50 nm. For all samples, the histograms obtained by DLS analysis do not show additional agglomeration. An example of DLS histograms is presented in Fig. 8. It is clearly seen that the size is monomodal and has a narrow distribution.

### Statistical Analysis

Statistical analysis was carried out to study the effect of varying synthesis parameters on the fluorescence properties and particle size obtained. The most desirable results are nanoparticles with the smallest possible size having at the same time strong light emission. The results for fluorescence excited with a light wavelength of 256 nm and 282 nm were analyzed.

### The Results of Dendrograms and K-means Clustering

As can be seen from the above studies, the obtained CDs respond to a different wave of excitation, which directly affects the fluorescence peak. It was decided to perform cluster analysis for CDs subjected to excitation at 256 nm and 282 nm. The analysis was based on the shape of the FL peak.

For further analysis of the results was also carried out. It allows assigning samples to a specific group - a cluster. Each individual cluster contains objects which are most similar to each other. In this case, objects with similar fluorescence properties were gathered in particular clusters.

#### Analysis for Excitation with λ=256 nm

**Fig. 9:**
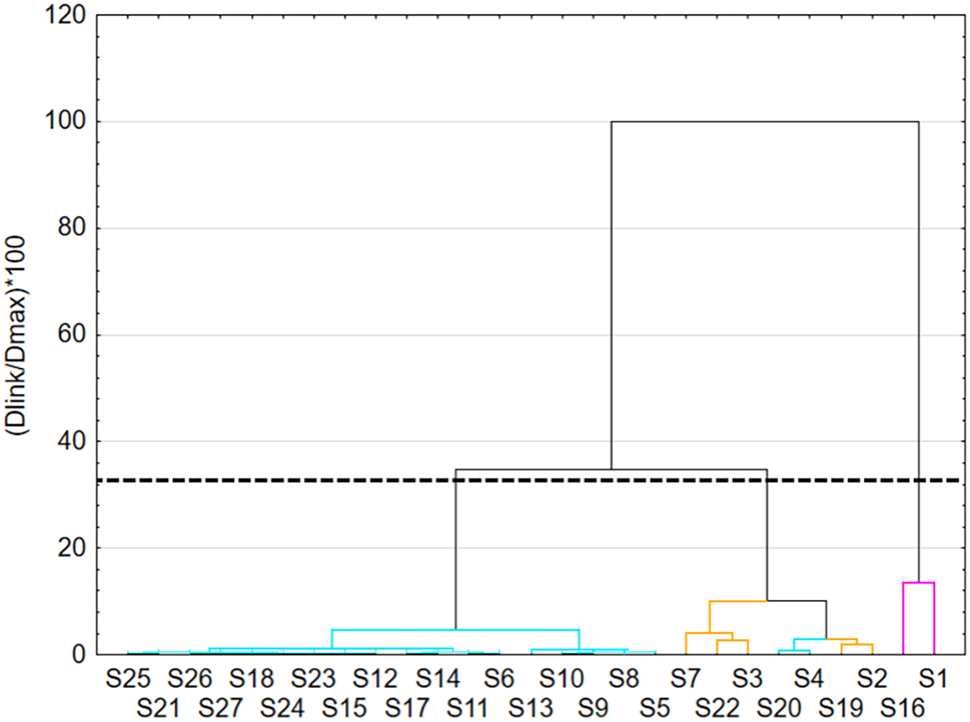
Hierarchical clustering dendrogram obtained using Ward’s method for FL intensity at 256 nm excitation wavelength

Results of cluster analysis are presented in the dendrogram made by Ward’s method with Euclidean distance measurements (Fig. 9). Ward's method involves grouping results into clusters using analysis of variance. Based on Sneath’s criterium in the diagram, three clusters can be distinguished. Cluster 1, which consists of only two samples, forms one cluster on the dendrogram. Cluster 2 consists of samples 2, 3, 4, 7, 19, 20, and 22. Cluster 3 forms a separate larger cluster. Samples from this cluster (left side of the dendrogram) have the best match.

**Fig. 10:**
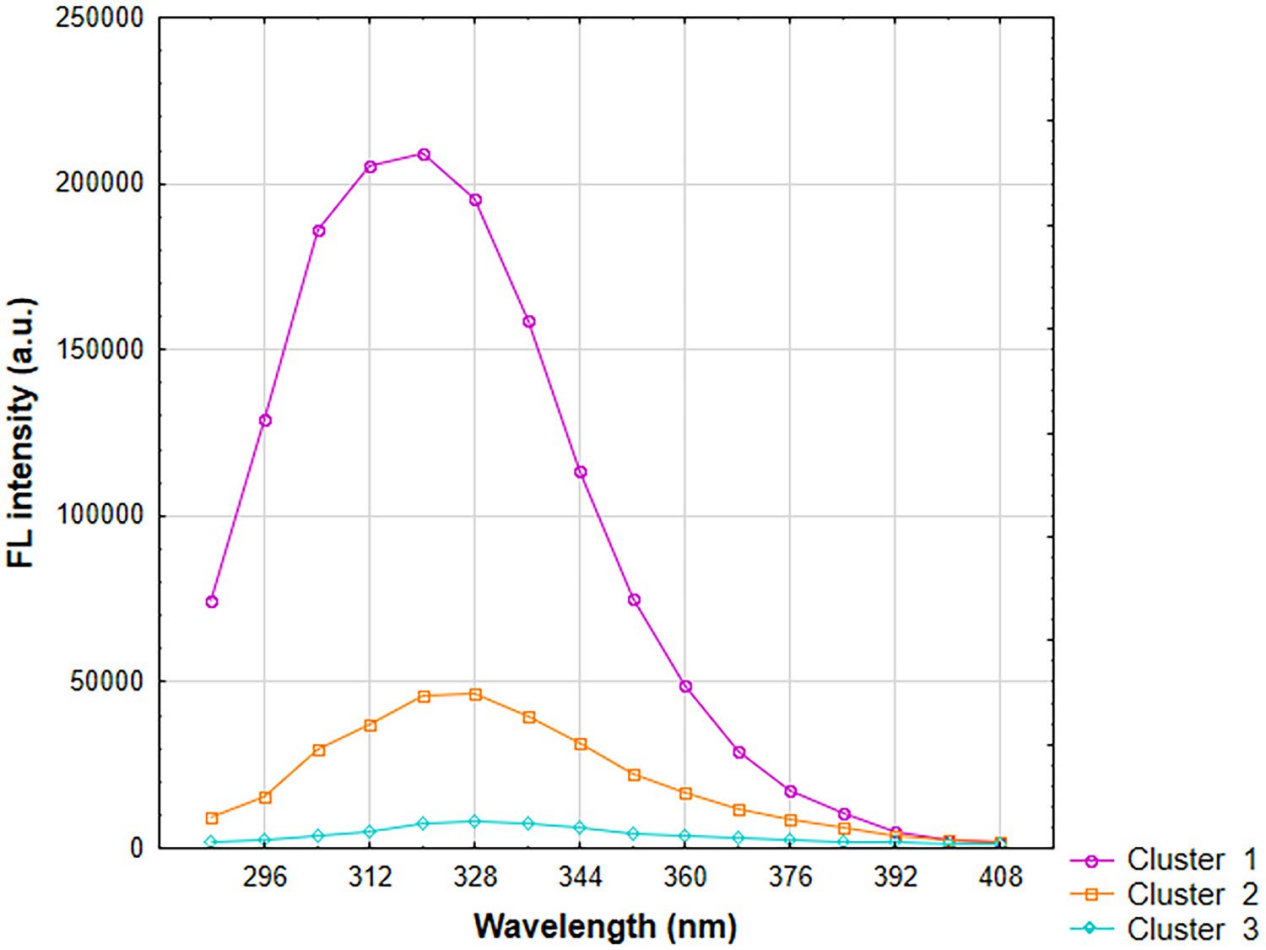
Results of the clustering of k-means for FL intensity at 256 nm excitation wavelength

A cluster analysis of the k-means of FL intensities at an excitation wavelength of 256 nm is shown in Figure 10. The analysis includes a wavelength range of 288-408 nm. In this case, a division into three clusters was also done, but there are significant differences in the graphs. The division of samples into clusters is shown in Table 2. The best results were obtained in the samples belonging to cluster 1. There are really only two samples - S1, and S16. In cluster 3, the FL intensity is negligible. In addition, it can be read from the graph that the peak maximum for samples from clusters 2 and 3 is at about 328 nm and for cluster 1 at about 320 nm of wavelength. The assignment of samples to clusters is similar to the hierarchical method from Figure 9. S4 and S20 samples were assigned to cluster 3 rather than cluster 2 as on the dendrogram. The remaining samples from the same clusters in both analyses.

**Table 2:** S-sample to clusters at 256 nm excitation wavelength.

**Cluster 1**	1	16																		
**Cluster 2**	2	3	7	19	22															
**Cluster 3**	4	5	6	8	9	10	11	12	13	14	15	17	18	20	21	23	24	25	26	27

These clusters can also be characterized based on the specific parameters used to perform their synthesis. In cluster 1, the common feature is P/G molar ratio equal to 0 and no homogenization time. In cluster 2, homogenization occurs at different times and most samples were obtained without potassium phosphate. Cluster 3 contains the remaining samples that are not matched anywhere, neither to cluster 1 nor to cluster 2. Thus, there is no dominant common feature.

Taking into account the results of DLS analysis and the assignment of samples to clusters, the best samples can be determined. S1 and S16 according to k-means analysis represent the samples with the most intense fluorescence. Their average size exceeds the preset 10 nm, giving sizes of 124 nm and 139 nm, respectively. In cluster 2, the most favorable samples are S3 (d=3 nm), S7 (d=8 nm) and S19 (d=3 nm). They are characterized by much lower fluorescence intensity than the samples in cluster 1. Therefore, the selection of the best samples depends on the established hierarchy of CDs properties. Strong fluorescence when samples are exposed to 256 nm light is obtained at the expense of particle size.

#### Analysis for Excitation with λ=282 nm

**Fig. 11:**
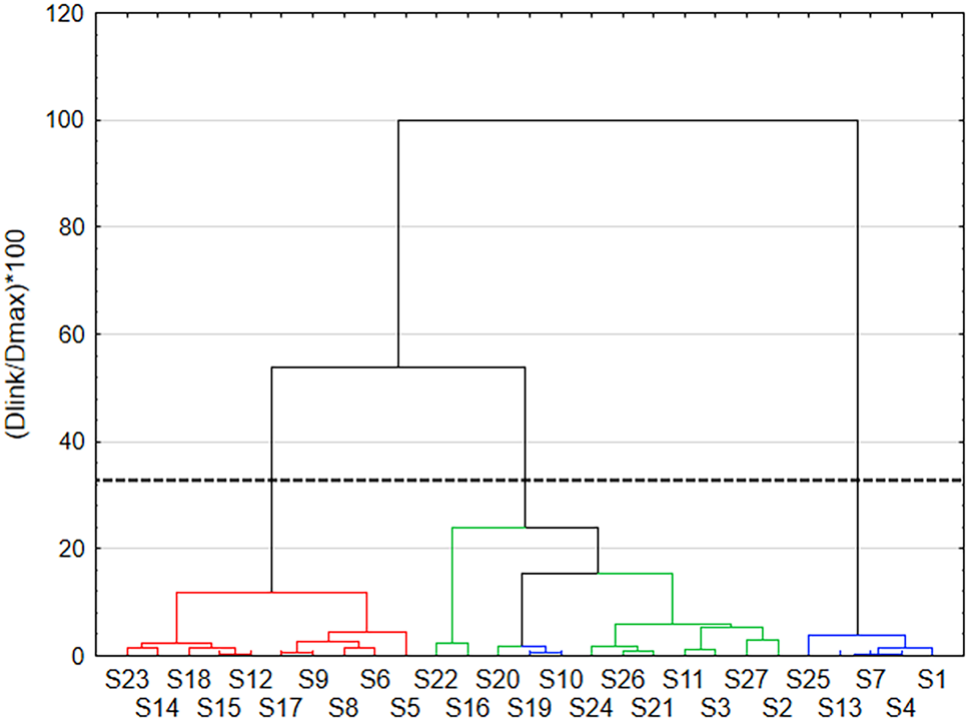
Hierarchical clustering dendrogram obtained with using Complete Linkage’s method for FL intensity at 282 nm excitation wavelength

In order to create a dendrogram for these results, the most appropriate method was Complete Linkage clustering and Squared Euclidean distance measurement (Fig. 11). The Complete Linkage method determines the distances between elements, looking for the best connections through cluster fusion. Commonly, this type of score prioritization is called farthest neighbor clustering. The graph clearly shows cluster membership and sample similarity. Colors were assigned based on both analyses to better illustrate the results and cluster membership of the samples.

The dendrogram shows two larger clusters. The best fit is between samples S12 and S15, S10 and S19, and between the three S4, S7, and S13. The restrictive Sneath criterion (33%) is drawn on the graph with a dotted line. It allows analysis of significant clusters, i.e. those below the cutoff line. This made it possible to distinguish three clusters. Each of them contains samples with similar properties.

**Fig. 12:**
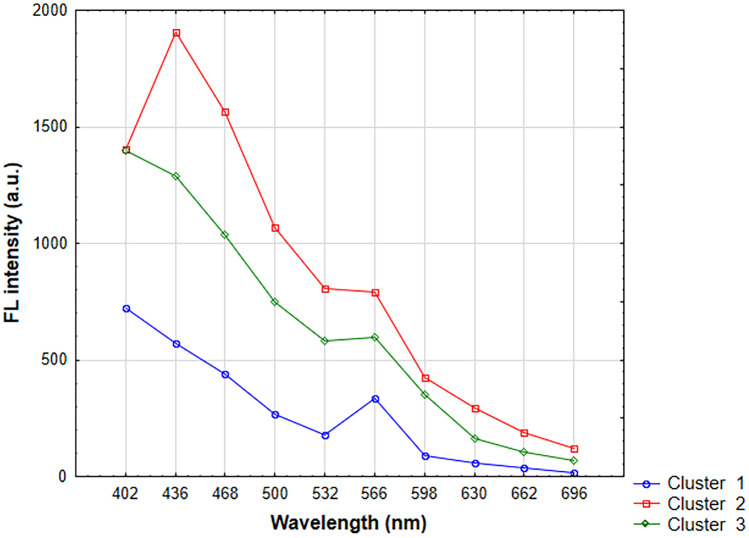
Results of the clustering of k-means for FL intensity at 282 nm excitation wavelength

Figure 12 shows a k-mean plot of individual clusters. The results were divided into three clusters (Tab. 3). Cluster 1 includes 7 samples. These were: S1, S4, S7, S10, S13, S19, and S25. It can be seen that the dominant variable parameter for that cluster is the P/G molar ratio which was equal to 0. These samples also showed weak or no FL after lamp irradiation. The highest FL intensity was achieved by samples from cluster 2. It contains samples 5, 6, 8, 9, 12, 14, 15, 17, 18, and 23. Ten samples whose syntheses were carried out at longer times (20, 38 min) were assigned to this cluster. Other common features that can be distinguished are lower glucose concentrations (0.01; 0.05 M), and the presence of KH_2_PO_4_. These samples have strong FL light in UV-lamp irradiation. Cluster 3 also contains 10 samples. These are: S2, S3, S11, S16, S20, S21, S22, S24, S26, and S27. Samples are characterized in that they were obtained at varied values of input parameters. Higher concentrations of glucose are preferred in this cluster. These results are consistent with cluster analysis using the hierarchical method. The exceptions are samples S10 and S19, which were assigned to samples from cluster 3 instead of cluster 1 as in the k-means method.

**Table 3:** Samples belonging to individual clusters when excited with 282 nm

**Cluster 1**	1	4	7	10	13	19	25			
**Cluster 2**	5	6	8	9	12	14	15	17	18	23
**Cluster 3**	2	3	11	16	20	21	22	24	26	27

The mean plot of cluster 1 has one maximum at 566 nm of wavelength. The graph of cluster 2 has two maximum at 436 nm and 566 nm. Cluster 3 has one maximum at 566 nm, but it is more intensive than in the case of cluster 1. It has also a strong background intensity between 402 and 500 nm. The graph of cluster 2 shows a strong peak at 436 nm. All graphs show weaker peaks at 566 nm.

Based on statistical analysis, it can be concluded that the particles from cluster 2 have the best properties. The highest fluorescence and the smallest sizes have CDs synthesized in S15 (d=9 nm), S5 (d=32 nm) and S12 (d= 33 nm). The difference in FL intensity between cluster 2 and 3 is not very high, so one can consider the results for samples from cluster 3, which have much more favorable particle sizes. In cluster 3, the best results were obtained for: S24 (d=1 nm), S3 (d=3 nm) and S11 (d=6 nm). S3 was determined to be the best sample in the k-means analysis for both 256 nm and 282 nm exposures. This sample fulfils the set criteria with high fluorescence and a particle size of less than 10 nm.

### Discussion of the Impact of Parameters

The effect of individual parameters on the resulting CDs was analyzed using Pareto charts. This method allows for prioritizing the factors that affect the synthesis product. Pareto charts show the type of relationship and the influence of a factor (the synthesis parameter analyzed) on the response based on the student’s t-test. The significance level (p) is the probability level of the test. The value is assumed to be 0.05 and is shown as a vertical red line in the graphs. It allows to separate statistically significant from non-significant parameters. The larger the value, the greater the contribution to the response, and thus the greater the impact on the obtained CDs properties.

**Fig. 13:**
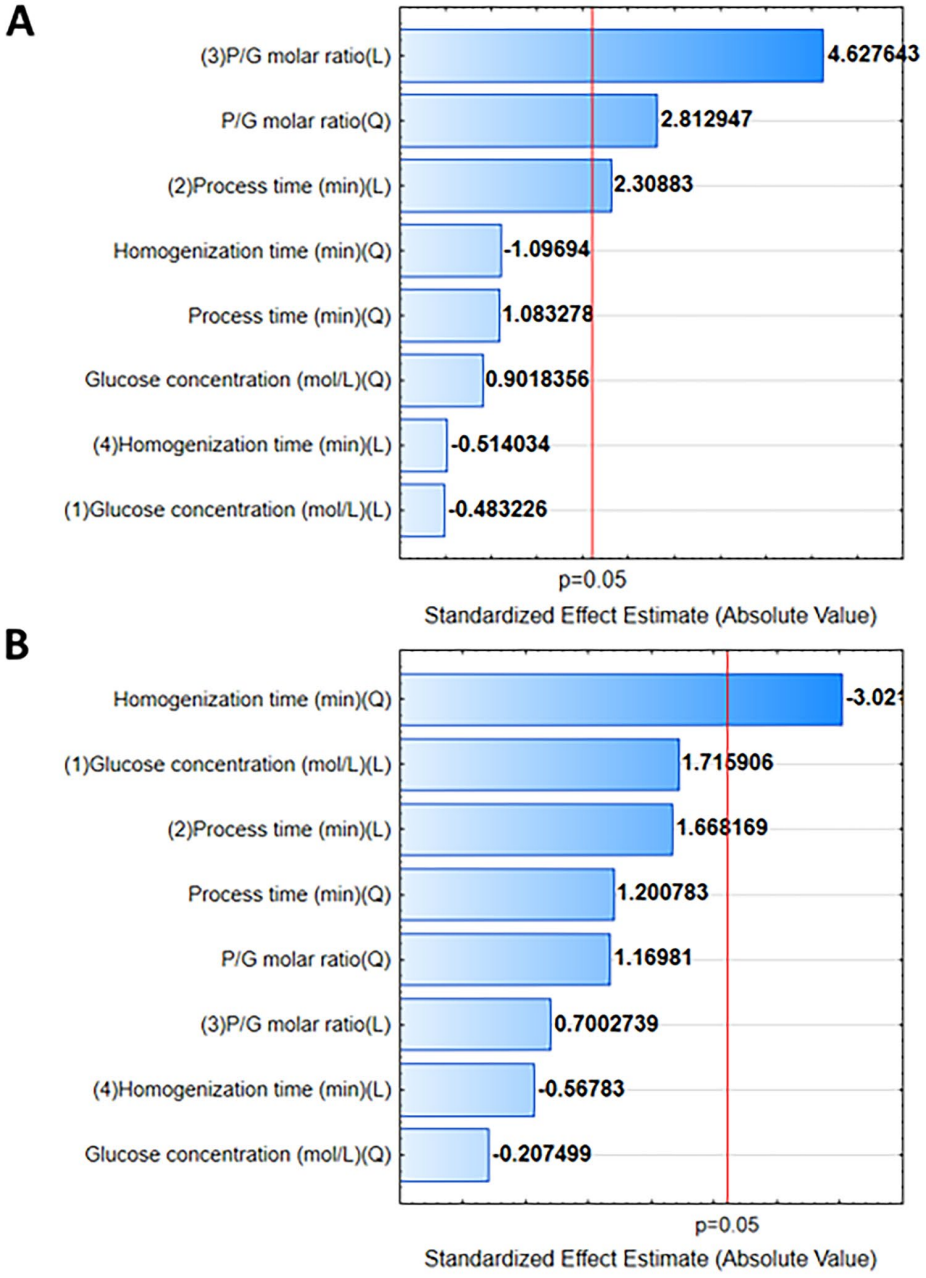
Pareto charts for(**A**) the area of the largest fluorescence peak at excitation light 282 nm, and (**B**) particle diameters

In the analysis of the spectrum in spectroscopic methods, various parameters of the peaks like height, width and peak area are important. In the presented study, the fluorescence peaks are quite broad without a clear maximum, especially in Figure 3. Therefore, it was decided to perform a statistical analysis based on the peak area. In this way, it is possible to trace the dependence of the parameters on the FL of the samples, which may have a wide low peak or a narrow high peak. Figure 13A shows Pareto plots for the results of fluorimetric analysis when CDs were exposed to 282 nm light. Additionally, values of particle diameters were analyzed if any input parameters affect them (Fig. 13B). The area under the peak for excitation light at 282 nm is affected by the P/G molar ratio in linear and quadratic functions. The results obtained are also influenced linearly by the processing time. Figure 13B shows that the size of the particles obtained in these syntheses was affected only by homogenization time in the quadratic function.

### Determination of Parameters That Allow Obtaining Desired Products

**Fig. 14:**
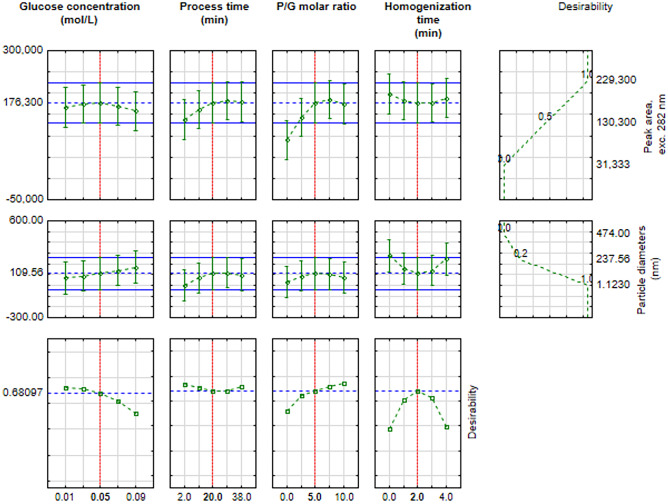
Profiles for predicted values and desirability functions for the peak area at 282 nm of excitation light and for particle diameters

In order to check the more specific influence of input the desirability profiling method was applied. Using different combinations of levels of independent variables, the desirability dependence functions for the dependent variables and their predicted values can be determined. The response profiles for the peak area under an excitation wavelength of 282 nm and particle diameters are summarized below (Fig. 14). Samples excited with 282 nm light cause secondary emission, but this peak has a much lower fluorescence intensity. Therefore, only the results of the first peak are considered for statistical analysis, without taking into account the secondary emission. The results for the excitation with 256 nm light were not included, as the certainty of the desired results was too low.

This representation of the results illustrates well whether the relationships are linear or quadratic. The most desired materials are those with alarger area peak of FL and size of the particles below 10 nm. In order to obtain such products, one should take the following process parameters: glucose concentration of 0.05 M, process time of 20 min, molar ratio of P/G 5 and homogenization time of 2 min. Using such parameters, it is possible to achieve this. Approximately 68% certainty that FL results at 282 nm excitation light and particle size will be in line with the predictions.

## Discussion

The above analyses show how important it is to select the right parameters influencing the desired parameters and properties of the CDs. Each parameter of the selected variables affected the resulting product. On this basis, CDs with different FL intensities at different excitation wavelengths were obtained.

The maximum intensity of CDs in UV-vis was the most influenced by the amount of KH_2_PO_4_ used and the glucose concentration. However, these parameters are statistically insignificant. The use of a dehydrating agent allows for an increase in the absorption of samples, which is directly proportional to the concentration of CDs in the solution. Similar UV-vis spectra were obtained in their study by researchers Z. Xu et al., J. Zheng et al. and C. Zhao et al. They indicate a high peak at around 280 nm and around 220 nm similar to Figs. 1 and 2. The synthesis methods were similar. The work of Z. Xu used H_3_PO_4_ as an additional reactant and resulted in carbon dots with yellow fluorescence. Using KH_2_PO_4_, the resulting suspension has yellow color and blue fluorescence which is also confirmed by the results of Z.-C. Yang [13, 21–23]. The blue fluorescence is also visible in the images in Fig. 6.

The study by Z.-C. Yang et al. indicate that the use of KH_2_PO_4_ should affect the size of the particles obtained. The researchers used glucose/KH_2_PO_4_ molar ratios of 1/26 and 1/36 in the synthesis. They obtained the smallest particles at the higher concentration of KH_2_PO_4_. The lack of use of this reagent resulted in up to a fivefold increase in particle size and aggregation of particles. The use of potassium phosphate also contributed to better fluorescence of the carbon dots. The researchers concluded that the concentration of the dehydrating agent could regulate the strength of fluorescence [23]. A similar relationship was also shown in the study analysis above. Depending on the wavelength of the excitation light, the KH_2_PO_4_ content was desirable or not. However, in this study, smaller particle sizes were obtained without potassium phosphate. For particles below 50 nm (as shown in Fig. 7), the dominant parameters were: a processing time of 2 min, a homogenization time of 2 min, and a P/G molar ratio of 0. However, it should be noted that the molar ratio used in synthesis was 5 or 10, so it may simply have been too low to obtain similar results as in the sample paper.

In the available studies, various wavelengths of light are used to expose CDs samples. This is mostly in the range of 300 to 500 nm. The dependence of the excitation wavelength on the FL emission intensity was presented by D. Stefanakis et al. shifting the excitation light wavelength by as much as 30 nm causes a large decrease in the intensity of emitted light by CDs [24]. They also managed to show, as the results obtained when irradiating the samples with 256 nm light are not equivalent to those at 282 nm. When CDs are irradiated with light at 256 nm, they emit light with a maximum intensity of 320 nm. When the sample of CDs is irradiated with a 282 nm wavelength, the maximum highest light emission is obtained at 440 nm and 560 nm. This state of matter is demonstrated in Figs. 3, 4, 5 at different excitation wavelengths. This result is not much different from the sample irradiation with a 330 nm light wave in the study of C. Zhao et al. or when irradiated with 354 nm light in the study of T. V. de Medeiros et al. A significant shift of the fluorescence peak is observed when excitation with a light wavelength of about 400 nm [22, 25].

The synthesis parameters show a linear and quadratic relationship with CDs fluorescence (Fig. 14). In the study, the main task was to achieve fluorescent particles. The FL peak area allowed for accurate FL analysis of the samples. This is an important parameter because the emission spectrum can vary. In this way, it is possible to trace the dependence of the parameters on the FL of the samples without excluding low but broad peaks. The most important parameters that have a significant impact on FL CDs are P/G molar ratio and process time.

Samples with high FL intensity at an excitation wavelength of 256 nm showed low FL intensity at 282 nm and vice versa. Interestingly, there is an inverse relationship. Samples with strong FL at 256 nm excitation wavelength were obtained without KH_2_PO_4_ and without homogenization (S1, S16). On the contrary, the best results were obtained with 282 nm excitation for samples S5, S6, S8, S9, S12, S14, S15, S17, S18, S23, which were obtained when the glucose concentration was lower, process time was longer and KH_2_PO_4_ was used. In order to obtain particles smaller than 50 nm, the desired parameters were: a processing time of 2 min, a homogenization time of 2 min and no KH_2_PO_4_. Surface matching indicates that the best parameters are a processing time of about 10 or 40 min and no homogenization. On the other hand, the particle size is expected to be as small as possible, and it can be achieved with a homogenization time of about 2 min and a low process time of up to 10 min. In the statistical analysis carried out, taking into account the fluorescence intensity under illumination with light of 256 nm and 282 nm and the particle size, the CDs sample from synthesis 3 was singled out as the best. S3 parameters were: glucose concentration 0.01M, process time 2 min, P/G molar ratio equal to 10, and homogenization time 2 min.

## Conclusions

Using the microwave synthesis method and aqueous reaction environment, it is possible to obtain CDs with a high FL intensity. By easily manipulating the process parameters, it is possible to influence the size of the obtained particles, as well as their optical properties. The presented studies show that the FL intensity depends on the many process parameters.

The excitation wavelength must be carefully and accurately selected for each CDs synthesis. The difference in the ability of particles to FL between excitation with 256 and 282 nm light is significant. This is influenced by the input parameters of the synthesis, which will translate into the number of CDs obtained and their structure. CDs obtained by the presented method show a UV-vis absorption maximum at 282 nm. Samples synthesized without KH_2_PO_4_ have a second peak with a UV-vis absorption maximum at about 230 nm. Depending on the excitation light length of the CDs, there is a shift in the emission peak. In both cases, the emission peak is broad. When the sample is irradiated with 256 nm excitation light, a peak is emitted in the 290-390 nm range with a maximum FL intensity of 320 nm. On the basis of statistical analysis, it was shown that the samples with the highest FL when irradiated with 256 nm light were those obtained without homogenization and without using KH_2_PO_4_. In the case of excitation with 282 nm light, two emission peaks with maxima of 440 and 560 nm, respectively, are visible. This time, longer synthesis time, lower glucose concentration, and the presence of KH_2_PO_4_ proved to be the best chosen parameters. However, taking into account such properties of the particles as their size and ability to FL the best parameters for 282 nm excitation turned out to be those with intermediate values: glucose concentration of 0.05 M, process time of 20 min, molar ratio of P/G 5 and homogenization time of 2 min. Using such reaction parameters makes it possible to obtain CDs with the smallest possible particle diameter and both the highest possible FL intensity with about 68% certainty.

In the syntheses performed, it can be deduced that better FL is obtained in longer processes, which unfortunately translates into an increase in particle size. A decisive influence on particle size is homogenization time. However, this particle parameter can be worsened with particle aggregation. It is recommended to choose a suitable separation method or add an agent to prevent particle aggregation. Further research is needed on CDs obtained according to the optimal reaction parameters from the analysis performed in this article. The effect of a parameter such as the pH of the environment should also be checked in future studies.

## Data Availability

The datasets used and/or analyzed during the current study are available from the corresponding author on reasonable request.

## References

[1] Liu J, Li R, Yang B (2020). Carbon Dots: A New Type of Carbon-Based Nanomaterial with Wide Applications. ACS Cent Sci.

[2] Lou Y (2021). Recent advances of biomass carbon dots on syntheses, characterization, luminescence mechanism, and sensing applications. Nano Select.

[3] Kurian M, Paul A (2021) Recent trends in the use of green sources for carbon dot synthesis–A short review. Carbon Trends 3:100032. 10.1016/j.cartre.2021.100032

[4] Bag P (2021). Recent Development in Synthesis of Carbon Dots from Natural Resources and Their Applications in Biomedicine and Multi-Sensing Platform. Chem Select.

[5] Ji C, Zhou Y, Leblanc RM, Peng Z (2020). Recent Developments of Carbon Dots in Biosensing: A Review. ACS Sens.

[6] Mohammadi R et al (2022) Fluorescence sensing and imaging with carbon-based quantum dots for early diagnosis of cancer: A review. J Pharm Biomed Anal 212:114628. 10.1016/J.JPBA.2022.114628 10.1016/j.jpba.2022.114628 35151068

[7] Zhang HC, Guo YM (2021). Advances of Carbon Quantum Dots for Fluorescence Turn-On Detection of Reductive Small Biomolecules. Chinese J Analytic Chem.

[8] Wang J et al (2021) Carbon dots based fluorescence methods for the detections of pesticides and veterinary drugs: Response mechanism, selectivity improvement and application. TrAC Trends Analytic Chem 144:116430. 10.1016/J.TRAC.2021.116430

[9] Wang B, Lu S (2022). The light of carbon dots: From mechanism to applications. Matter.

[10] Yan F, Sun Z, Zhang H, Sun X, Jiang Y, Bai Z (2019) The fluorescence mechanism of carbon dots, and methods for tuning their emission color: a review. Microchimica Acta 186(8). 10.1007/s00604-019-3688-y 10.1007/s00604-019-3688-y 31359150

[11] Liu M (2020). Optical Properties of Carbon Dots: A Review. Nanoarchitectonics.

[12] Yoshinaga T, Iso Y, Isobe T (2018). Particulate, Structural, and Optical Properties of D-Glucose-Derived Carbon Dots Synthesized by Microwave-Assisted Hydrothermal Treatment. ECS J Solid State Sci Technol.

[13] Xu Z, Wang C, Jiang K, Lin H, Huang Y, Zhang C (2015). Microwave-Assisted Rapid Synthesis of Amphibious Yellow Fluorescent Carbon Dots as a Colorimetric Nanosensor for Cr(VI). Particle, Particle Syst Character.

[14] Liu Y, Huang H, Cao W, Mao B, Liu Y, Kang Z (2020). Advances in carbon dots: from the perspective of traditional quantum dots. Mater Chem Front.

[15] Ma W, Wang B, Yang Y, Li J (2021). Photoluminescent chiral carbon dots derived from glutamine. Chinese Chem Lett.

[16] Lai S, Jin Y, Shi L, Zhou R, Zhou Y, An D (2020). Mechanisms behind excitation- and concentration-dependent multicolor photoluminescence in graphene quantum dots. Nanoscale.

[17] Choi YJ, Sawada K (2023). Physical Sensors: Fluorescence Sensors, Encyclopedia of Sensors and Biosensors: Volume 1–4. First Edition.

[18] Itagaki H (2000) Fluorescence Spectroscopy, Experimental Methods in Polymer Science: Modern Methods in Polymer Research and Technology, pp. 155–260. 10.1016/B978-0-08-050612-8.50009-X

[19] Carbonaro CM (2018). Carbon Dots in Water and Mesoporous Matrix: Chasing the Origin of their Photoluminescence. J Phys Chem C.

[20] Xu Y, Wang C, Ran G, Chen D, Pang Q, Song Q (2021). Phosphate-Assisted Transformation of Methylene Blue to Red-Emissive Carbon Dots with Enhanced Singlet Oxygen Generation for Photodynamic Therapy. ACS Appl Nano Mater.

[21] Zheng JX, Liu XH, Yang YZ, Liu XG, Xu BS (2018). Rapid and green synthesis of fluorescent carbon dots from starch for white light-emitting diodes. Xinxing Tan Cailiao/New Carbon Mater.

[22] Zhao C, Li X, Cheng C, Yang Y (2019). Green and microwave-assisted synthesis of carbon dots and application for visual detection of cobalt(II) ions and pH sensing. Microchem J.

[23] Yang ZC (2011). Intrinsically fluorescent carbon dots with tunable emission derived from hydrothermal treatment of glucose in the presence of monopotassium phosphate. Chem Commun.

[24] Stefanakis D, Philippidis A (2014) Synthesis of fluorescent carbon dots by a microwave heating process : structural characterization and cell imaging applications. 10.1007/s11051-014-2646-1

[25] De Medeiros TV, Manioudakis J, Noun F, Macairan JR, Victoria F, Naccache R (2019). Microwave-assisted synthesis of carbon dots and their applications. J Mater Chem C Mater.

